# Genome survey of resistance gene analogs in sugarcane: genomic features and differential expression of the innate immune system from a smut-resistant genotype

**DOI:** 10.1186/s12864-019-6207-y

**Published:** 2019-11-06

**Authors:** Hugo V. S. Rody, Renato G. H. Bombardelli, Silvana Creste, Luís E. A. Camargo, Marie-Anne Van Sluys, Claudia B. Monteiro-Vitorello

**Affiliations:** 1 Escola Superior de Agricultura “Luiz de Queiroz”, Departamento de Genética, Universidade de São Paulo, Piracicaba, São Paulo, Brazil; 2 Centro de Cana, IAC-Apta, Ribeirão Preto, Av. Pádua Dias n11, CEP 13418-900, Piracicaba, São Paulo, Brazil; 3 Departamento de Botânia, Universidade de São Paulo, Instituto de Biociências, São Paulo, Brazil

**Keywords:** *Sporisorium scitamineum*, *Saccharum*, Crop, Disease resistance

## Abstract

**Background:**

Resistance genes composing the two-layer immune system of plants are thought as important markers for breeding pathogen-resistant crops. Many have been the attempts to establish relationships between the genomic content of Resistance Gene Analogs (RGAs) of modern sugarcane cultivars to its degrees of resistance to diseases such as smut. However, due to the highly polyploid and heterozygous nature of sugarcane genome, large scale RGA predictions is challenging.

**Results:**

We predicted, searched for orthologs, and investigated the genomic features of RGAs within a recently released sugarcane elite cultivar genome, alongside the genomes of sorghum, one sugarcane ancestor (*Saccharum spontaneum*), and a collection of de novo transcripts generated for six modern cultivars. In addition, transcriptomes from two sugarcane genotypes were obtained to investigate the roles of RGAs differentially expressed (RGADE) in their distinct degrees of resistance to smut. Sugarcane references lack RGAs from the TNL class (Toll-Interleukin receptor (TIR) domain associated to nucleotide-binding site (NBS) and leucine-rich repeat (LRR) domains) and harbor elevated content of membrane-associated RGAs. Up to 39% of RGAs were organized in clusters, and 40% of those clusters shared synteny. Basically, 79% of predicted NBS-encoding genes are located in a few chromosomes. *S. spontaneum* chromosome 5 harbors most RGADE orthologs responsive to smut in modern sugarcane. Resistant sugarcane had an increased number of RGAs differentially expressed from both classes of RLK (receptor-like kinase) and RLP (receptor-like protein) as compared to the smut-susceptible. Tandem duplications have largely contributed to the expansion of both RGA clusters and the predicted clades of RGADEs.

**Conclusions:**

Most of smut-responsive RGAs in modern sugarcane were potentially originated in chromosome 5 of the ancestral *S. spontaneum* genotype. Smut resistant and susceptible genotypes of sugarcane have a distinct pattern of RGADE. TM-LRR (transmembrane domains followed by LRR) family was the most responsive to the early moment of pathogen infection in the resistant genotype, suggesting the relevance of an innate immune system. This work can help to outline strategies for further understanding of allele and paralog expression of RGAs in sugarcane, and the results should help to develop a more applied procedure for the selection of resistant plants in sugarcane.

## Background

Plants have evolved a two-layer immune system in order to hamper pathogen attacks [1, 2]. Resistance signaling cascades are triggered in the plants throughout direct/indirect association of their resistance genes with either the pathogen-associated molecular patterns (PAMPs) — first layer, the PAMP-Triggered Immunity (PTI) — or with specific effectors — second layer, the Effector-Triggered Immunity (ETI) [1]. Consequently, the genomic content of Resistance Gene Analogs (RGAs) is frequently associated with crop resistance and have been gathering the attention of many breeding programs [3–5]. RGAs have conserved domains/motifs and structural features, and can be classified into two major encoding families: 1) the classical R genes harboring a nucleotide-binding site followed by leucine-rich repeat (NBS-LRR or NLRs); and 2) the pattern recognition receptors (PRR) characterized by transmembrane domain followed by leucine-rich repeat (TM-LRR) [2]. RGAs also have a notably genomic organization. Both the classical genetics [6] and analysis from large scale sequencing data [3] have shown RGAs biased to form clusters in the plant genomes. These clusters may contain RGAs related in function but not necessarily in sequence [7]. Ancient whole-genome duplications (WGDs), in addition to segmental duplications, both followed by gene deletions and genomic reorganizations have contributed to the expansion of RGA families [8, 9].

Based on the conserved structural characteristics of RGAs, genomic screening approaches may represent an important strategy for breeding pathogen-resistant crops. Sugarcane (*Saccharum* spp.) is one of the most economically important crops, responsible for 80% of total sugar produced in the world (“European Commission of Agriculture and rural development. Sugar.,” n.d.). Sugarcane plantations are often opposed by diseases that culminate in economic losses. Many attempts have been made to establish relationships between the RGA content of modern sugarcane cultivars to its degrees of resistance to diseases caused by pathogens such as rust [10–12], yellow leaf [13], red hot [14–17], and smut [18–21]. The strategies applied to investigate RGAs in sugarcane have mainly focused on the development of degenerate primers targeting conserved RGA motifs [15, 16, 22], in addition to the structural identification from expressed sequence tag (EST) libraries [10–12, 14, 20].

The ploidy and highly repetitive genome characteristics of sugarcane have imposed challenges for breeding. Modern sugarcane cultivars are products from hybridizations between *S. officinarum* L. and *S. spontaneum* L. [23]. The domesticated *S. officinarum* L. (2n = 80) was used because of its high sugar content, whereas the wild *S. spontaneum* L. (2n = 40 to 128) was expected to bring disease resistance. Genomic references have been recently released for sugarcane. A sugarcane monoploid genome from the elite cultivar R570 was achieved [24] from the alignment of cloned inserts in bacterial artificial chromosomes (BAC) to the *Sorghum bicolor* genome. Shortly after, the genome of one important autopolyploid ancestor of sugarcane, the tetraploid *S. spontaneum* L. clone of SES208 namely AP85–441 was also published [25]. The release of aforementioned genomes makes feasible new genomic research in sugarcane. Investigation of the RGA content within those genomes may shed light on the molecular basis of sugarcane resistance to diseases. The sugarcane smut disease, for example, is spread worldwide and during severe infections may result in production losses up to 62% [26, 27]. Smut is caused by the biotrophic fungus *Sporisorium scitamineum* and is mainly characterized by the development of a whip-like structure from the primary meristems. As could be anticipated from biotrophic fungi, no hypersensitive response has been reported during the smut-sugarcane interaction. Although oxidative burst in the early stages of infection has been shown for smut-resistant sugarcane cultivars [28], no genomic investigation has focused on the investigation of RGAs involved in the first layer of sugarcane immune system. Herein, we used conserved structural features to predict RGAs in three references of sugarcane for comparative analysis: the monoploid genome of the modern sugarcane cultivar R570 [24], a monoploid version of the genome of sugarcane ancestor *S. spontaneum* AP85–441 [25], and a broad set of de novo unique transcripts (*N* = 88.488) generated from data of six modern sugarcane cultivars, including the RB925345 that has been obtained after inoculation with smut [21, 29]. In addition, we also analized RGAs within the genome of *Sorghum bicolor* [30], a genome reference commonly used for sugarcane comparative analysis. We then analyzed the transcriptome profiles from two modern sugarcane genotypes — having distinct degrees of resistance to smut disease — to investigate the early stages of RGA expression during smut-sugarcane interaction. In particular, we addressed the following questions: 1) How many RGAs can be predicted within the genomes of sugarcane ancestors, and within the available genome of modern sugarcane cultivar? 2) How are they distributed and organized within those genomes? 3) Do transcriptomes from sugarcane genotypes having distinct degrees of resistance to smut can help to unravel the roles of PTI and ETI immune systems during the early stages of sugarcane-smut interaction? 4) Do the orthologs of differentially expressed RGAs are biased towards chromosomes, clusters, or syntenic segments? 5) Do their expression profiles reflect their phylogenetic relationships?

## Results

Our strategy was first to develop a pipeline to retrieve and classify RGAs in the protein of four sugarcane references: 1) the available monoploid genome versions of the sugarcane cultivar R570, and 2) *S. spontaneum* AP85–441, 3) the genome of *Sorghum bicolor*, in addition to 4) a set of de novo unique transcripts assembled from RNAseq data from six modern sugarcane cultivars. We then established the genome organization of predicted RGAs in the two sugarcane genomes and *S. bicolor*, followed by a phylogenetic study. Finally, a transcriptomic approach revealed the differential expression profile of the RGAs using two sugarcane cultivars with different degrees of smut susceptibility.

### Prediction of RGAs and database assembly

We used a set of five softwares to search for conserved RGA domains in the protein sequences within four focal sugarcane references (see methods). Custom Python3 scripts were then used to parse the predictions outputs from the five softwares and to classify the sequences as RGAs according to the combination of domains predicted (see methods). During validation, our pipeline succeeded in predicting conserved RGA domains for the majority (~ 97%) of the R reference genes from the PRG database [31] (Additional file 1). Out of 128 R reference genes from PRGdb, only four genes had no RGA-related domains predicted. The presence of transmembrane domains (TM) was the most frequent divergence among the annotation retrieved from PRGdb and our pipeline predictions. Nine PRGdb protein sequences were not initially considered as RGA because they lacked essential RGA domains combinations, or some of the used softwares failed during predictions. Additionally, protein sequences were also analyzed using orthology relationships via BLAST searches against R reference orthologs from PRGdb (Additional file 2). The largest part of RGAs (> 62%) predicted as R orthologs had at least one conserved RGA domain previously predicted by our pipeline, but were firstly considered as non-RGA because they lacked RGA combination of domains previously described (see methods).

Five classes of RGAs were more frequently predicted within the four focal references of this study:1) CN: coiled coil (CC) domain associated to NB-ARC; 2) CNL: CC associated to NB-ARC and leucine-rich repeats (LRR); 3) RLK: Receptor-like kinase; 4) RLP: Receptor-like protein; and 5) TM-CC: Transmembrane domain associated to CC (Table 1 The TNL class, TIR domain associated to NB-ARC and LRR, from the NBS-LRR encoding family, was not predicted. RGAs harboring other domains combinations than those five aforementioned represented up to 11%. The two classes of RGAs associated to cell membranes of TM-CC and RLK presented the most significant number of RGAs predicted.

### Sugarcane genomic organization of RGAs, orthology, clusters, and synteny

**Fig. 1:**
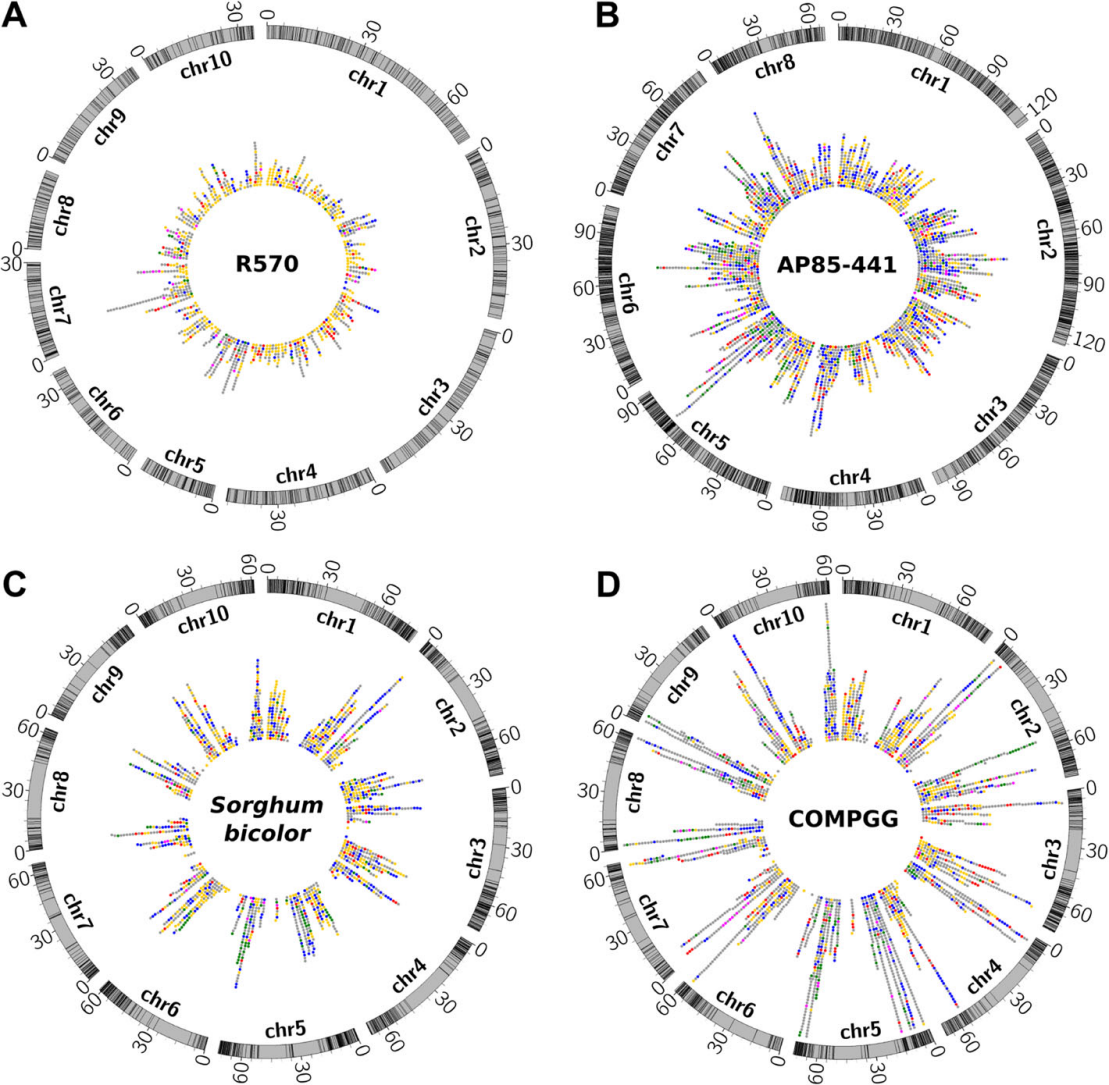
Distribution of RGAs predicted within four sugarcane references along their respective genomes. **a** RGAs predicted for R570 sugarcane cultivar distributed along its 10 chromosomes monoploid genome. **b** RGAs predicted for AP85–441 *S. spontaneum* distributed along its eight chromosomes of its monoploid genome. **c** RGAs predicted for *S. bicolor* distributed along its 10 chromosomes. **d** RGAs predicted for COMPGG de novo transcript sequences distributed along 10 chromosomes of *Sorghum bicolor*. Rings indicate the chromosomes in Mbp. Traces in chromosomes indicate RGAs positions. Colored dots indicate RGAs according to classes: CN: purple; CNL: green; RLK: blue; RLP: red; TM-CC: yellow; Other variants: grey

Genomic coordinates of RGAs from the three genomic references (cultivar R570, *S. spontaneum* AP85–441, and sorghum) were used to investigate their organization. For the sequences from the COMPGG dataset, we attributed genomic coordinates from sorghum sequences based on best hits BLASTp searches (see methods). The predicted RGAs were found distributed along all the chromosomes within each of the four targeted references of this study (Fig. 1). Sorghum presented the smallest percentage of RGAs having chromosome annotations. From the total of 1919 RGAs predicted for sorghum, 1449 (75.5%) were found within chromosome. The AP85–441 had the largest percentage, were 2337 out of the total of 2354 RGAs predicted (> 99%).

Also, RGAs in sorghum were arranged differently from both R570 and AP85–441 (Fig. 1b-d). They were more frequently positioned at the extremities of the chromosomes (Fig. 1d) — away from centromeric regions —, whereas in sugarcane references the RGAs were evenly distributed over the chromosomal extension (Fig. 1b,c).

COMPGG dataset showed longer sequences of dots as depicting RGAs across the chromosomes of sorghum genome (Fig. 1b). Similarly, a few other long sequences of dots were present in the genomes of AP85–441 (chromosomes 4, 5, 6, 7, and 8), R570 (chromosomes 5 and 7), and sorghum (chromosomes 2, 5 and 10).

We addressed RGA organization as single, two or organized in clusters (see methods) for the three genomes references (Table 2). Clusters span regions from > 8 Kbp to < 743 Kbp, with sorghum harboring the shortest and AP85–441 harboring the largest cluster. In both the sorghum and R570 genomes, the chromosomes 5 and 2 accommodate the largest number of RGA clusters. Sorghum genome had the largest number (*N* = 179) of predicted RGA clusters, whereas the R570 had the smallest number (*N* = 79). The sorghum genome also had the largest percentage (39%, *N* = 749) of RGAs organized in clusters, followed by R570 (31%; *N* = 308), and the genome of AP85–441 with the smallest percentage (23%; *N* = 556) (Additional file 2). In the genome of *S. spontaneum* AP85–441, were the chromosomes 6 (Ss6) and 2 (Ss2) those sheltering the largest number of RGA clusters; 25 clusters in each of the two chromosomes (Additional file 2). The largest number of RGAs in a single cluster (*N* = 17) was encountered within the chromosome Ss4 of AP85–441 genome. This large RGA cluster span from about 55 Kbp and consisted of 8 TM-LRR sequences (5 RLKs and 3 RLPs), together with 9 more RGAs harboring other domains combinations.

Many of the RGAs predicted as organized in clusters were also predicted as originated from tandem duplications events. In sorghum, ~ 62% of the cluster-arranged RGAs were also predicted by the DAGchainer software as tandem-derived. The sugarcane genomic references AP85–441 and R570 had ~ 48% and ~ 46%, respectively, of their cluster-arranged RGAs also predicted as tandem-derived.

The OrthoMCL software predicted a total of 1459 orthogroups containing at least one of predicted RGAs. Were 220 RGA orthogroups harboring at least one RGA from each of the four references (*N* = 2736 RGAs), which comprises more than 35% of the total of RGAs (*N* = 7703) predicted (Additional file 2; Additional file 3: Figure S6a).

From the total of 2736 RGAs found within the 220 orthogroups mentioned above, 675 were transcripts from COMPGG. Therefore, we predicted synteny and clusters for 2061 RGAs. Out of these 2061 RGAs, 720 (35%) were also found within syntenic segments, and more than 47% (*N* = 341 of 720) were also found forming clusters.

**Fig. 2:**
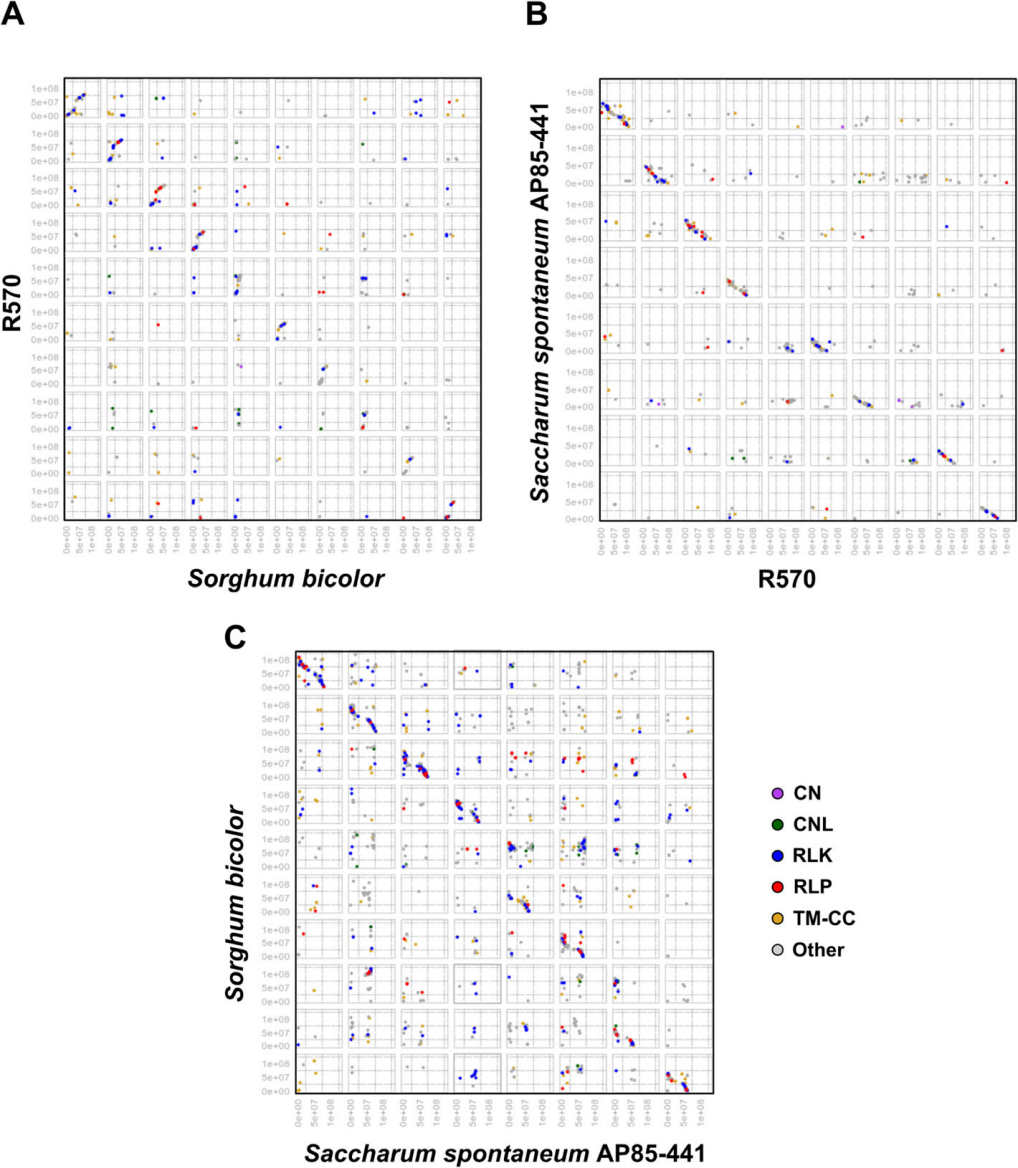
Shared synteny dot plots among predicted RGAs from three sugarcane reference genomes. Dots represents gene pairs alignments identified by DAGchainer software for: **a** R570 and *S. bicolor*. **b** AP85–441 and R570. **c** *Sorghum bicolor* and AP85–441. Axis show chromosomes coordinates in base pairs

We used DAGchainer to investigate shared synteny among the three focal genome references. Thus, synteny was firstly evaluated considering the complete set of proteins sequences encoded from each genome and reported for segments containing at least 12 genes arranged in pairs (six pairs). Sorghum genome had the largest number (*N* = 8899) of genes found within syntenic segments, whereas the R570 genome presented the lowest number of genes in synteny (*N* = 5594). A total of 2907 syntenic segments were found among the three references, with the longest segment (189 gene pairs) identified between the chromosome Sb10 of sorghum and the chromosome Ss8 of AP85–441 (Fig. 2; Additional file 2). RGAs were amongst the genes identified by the DAGchainer as sharing synteny (Fig. 2; Additional file 2). Several syntenic segments harboring RGAs were observed for the alignments performed between AP85–441 and sorghum genomes (Fig. 2a), and between AP85–441 and R570 (Fig. 2b). Shorter syntenic fragments were also identified in the alignments between R570 and sorghum (Fig. 2c). About 54% of RGAs identified within the AP85–441 genome (Table 1) (*N* = 611 of 2353) were located in syntenic segments, followed by 28% (*N* = 538 of 1917) of sorghum RGAs, and 27,5% (*N* = 264 of 960) of RGAs predicted within the R570 genome.

**Table 1:** Number of predicted RGA candidates by encoding families of nucleotide-biding site followed by leucine-rich repeat (NBS-LRR) and transmembrane domain followed by LRR (TM-LRR) and their classes within each of the four targeted sugarcane references of this study

RGA class	Reference			
	R570	AP85–441	*S. bicolor*	COMPGG
*NBS-LRR encoding*	47	137	139	109
CNL	22	154	135	140
TNL	0	0	0	0
*TM-LRR encoding*				
RLK	79	427	404	290
RLP	60	157	100	154
*Other variants*				
TM-CC	313	450	482	307
CN	21	36	21	64
NBS-encoding	53	75	38	257
LRR-encoding	336	635	389	998
Other combinations	29	282	209	151
Total number of RGAs	960	2354	1919	2470

**Table 2:** Overview of clusters of RGAs predicted within three genome references of sugarcane

Statistics	R570	AP85–441	*S. bicolor*
Total number of clusters	79	136	179
Total number of RGAs arranged in clusters	308	556	749
Largest number of RGAs in a cluster	10	17	11
Maximum cluster length (bp)	359,057	742,308	570,975
Maximum number of RLKs in a cluster	2	5	7
Maximum number of RLPs in a cluster	4	5	4
Maximum number of CNLs in a cluster	1	6	7
Maximum number of TM-CC in a cluster	4	4	4

We detected synteny amongst the RGAs found within clusters. On average, 40% of the RGAs within clusters were also within syntenic blocks. The total number of cluster-arranged RGAs in syntenic segments regions were 259 in sorghum, 215 in AP85–441, and 109 in the R570 genome. The chromosomes harboring the largest number of cluster-arranged RGAs sharing synteny were chromosome Ss6 from AP85–441 (67 RGAs), chromosome Sb5 from sorghum (46 RGAs), and chromosome Sh7 from R570 (23 RGAs).

The syntenic segments from Sb5 and Ss6 chromosomes were from the classes of RLK and CNL (Additional file 3: Figure S2). RLP and TM-CC were also found within short fragments of synteny. RLPs were syntenic between chromosomes Sb10 and Ss8, and TM-CCs shared synteny between Sb10 and Sh10 (Additional file 3: Figure S2).

### Transcriptome analysis of two sugarcane genotypes inoculated with smut

Transcriptome profiles from the two sugarcane varieties of SP80–3280 (smut-resistant) and IAC66–6 (smut-susceptible) were obtained to investigate differential expression of RGAs during an initial stage of smut disease. RNAseq data were obtained for 12 libraries: from each of the two genotypes, were three biological replicates for control plant buds, and three replicates for buds 48 h after inoculation (hai) with the *S. scitamineum* (SSC39). From the ~ 105 million paired-end sequence reads (~ 8 million reads per library) obtained, more than 97% were kept after the preprocessing step (see methods) (Additional file 3: Table S1).

We used the COMPGG dataset as reference for the assembly of the reads because it represents the largest published collection of transcripts obtained for modern sugarcane varieties. Out of the 88,488 COMPGG total transcript sequences, more than 69 thousand sequences (~ 76%) were assembled within each library. Transcriptome assembly of control plants generated 72,078 transcripts for IAC66–6 as compared to 69,356 assembled transcripts for the smut-resistant genotype, SP80–3280. Control plant libraries had a particular number of uniquely assembled sequences between the two genotypes. The smut-susceptible IAC66–6 control plants had 6922 uniquely assembled sequences, whereas the smut-resistant SP80–3280 control plant had 4200 (Additional file 2). Differences in the number of uniquely assembled sequences between sugarcane genotypes were also observed for inoculated plants. The smut-susceptible genotype inoculated plants had 4879 sequences exclusively assembled, whereas the smut-resistant genotype inoculated plants had 7508. During smut-sugarcane interaction, the total number of transcripts considered as expressed in the smut-susceptible genotype was 40,248, whereas in the smut-resistant was 38,441. Resistant and susceptible genotypes shared 36,006 expressed transcripts when interacting with smut.

The total number of Differentially Expressed Genes (DEGs, inoculated/control) were different among sugarcane genotypes. The IAC66–6 smut-susceptible genotype had 2300 DEGs, whereas the smut-resistant SP80–3280 had 3440. Only 200 DEGs were in common among sugarcane genotypes.

**Fig. 3:**
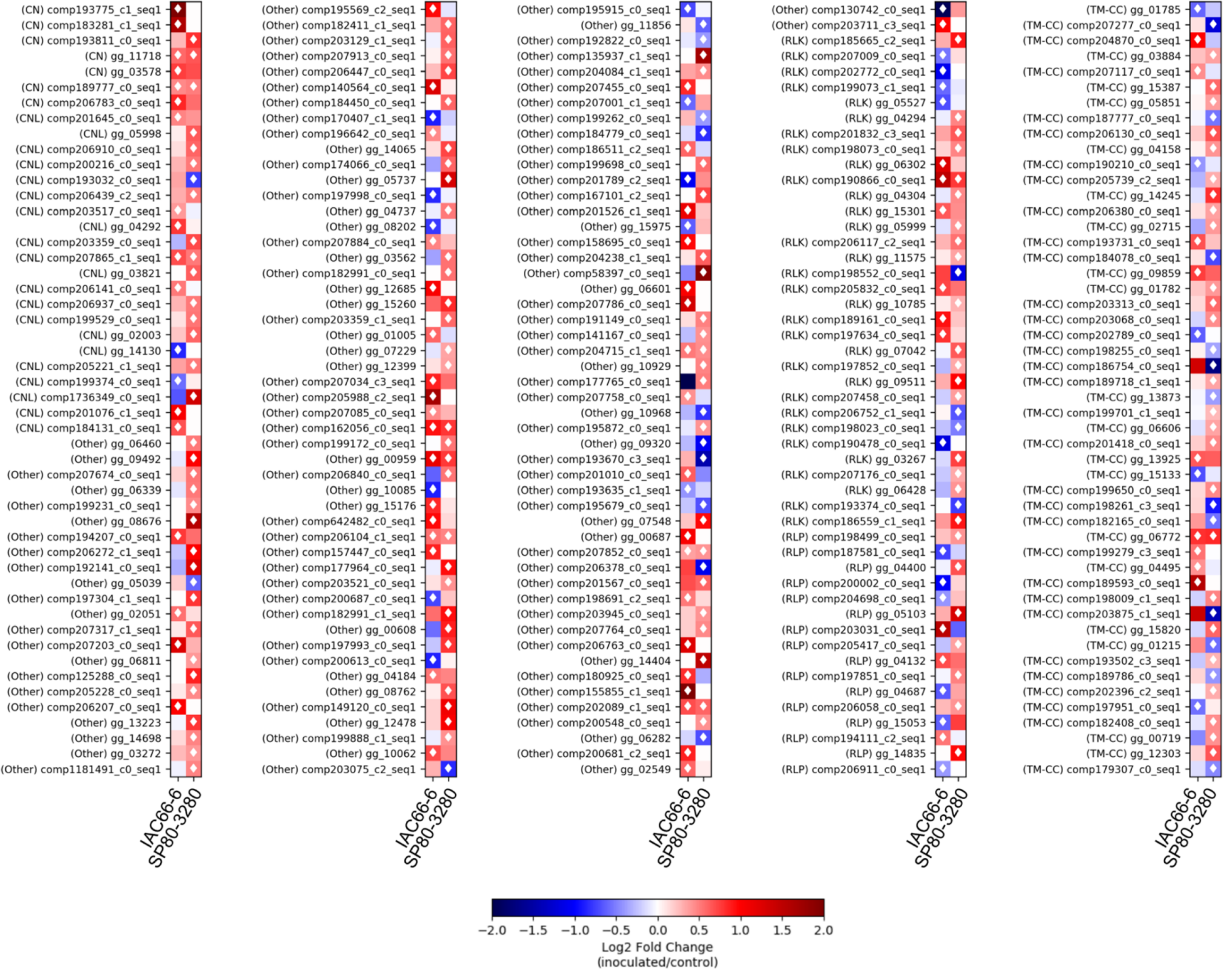
Expression profile of 250 RGAs predicted within two sugarcane genotypes with contrasting degrees of resistance to smut. Transcripts were assembled having COMPGG dataset as reference, and expression is represented as Log2 Fold Change values (inoculated/control). Blue squares represent down-regulation, whereas red squares represent up-regulation. Black squares represent no transcript expression. The statistical significance of expression is presented in Additional file 2

RGAs were amongst the predicted DEGs (Fig. 3). Hereinafter, we will report to them as RGADE. From the total of 101 RGADE found within IAC66–6 genotype, 90 were unique. In the SP80–3280 genotype 149 were unique from the total of 160. The two targeted genotypes shared only 11 RGADE. Out of 11 RGADE shared between sugarcane genotypes, one fell into each of the CNL, RLK and TM-CC classes, two were predicted as CN, and six harbored different domain combinations. No RGADEs from RLP class were found shared by sugarcane genotypes. The smut-susceptible genotype of IAC66–6 presented 20 RGADE from TM-LRR encoding family: 11 from RLK class, and nine from the RLP. Compared to the susceptible genotype of IAC66–6, the SP80–3280 smut-resistant genotype presented more RGADE (*N* = 29) from TM-LRR: 22 RLKs, and 7 RLPs. The TM-CC class of RGAs had the highest number of RGADEs: were 14 within IAC66–6 and 37 within SP80–3280. The expression of CNL was found very distinct between the two sugarcane genotypes. Although most of CNL were significantly up-regulated in sugarcane genotypes, only one single up-regulated CNL (comp207865_c1_seq1) was shared between the genotypes.

We additionally investigated the RGADE expression profile of the two targeted sugarcane genotypes at the ortholog groups (orthogroups) level. Most of RGADE orthogroups from IAC66–6 and SP80–3280 were distinct. Out of 101 RGADE predicted within the IAC66–6, 71 RGADE were found as composing 45 different orthogroups, whereas 30 RGADE did not form any orthogroup. Within the SP80–3280 genotype, out of 160 predicted RGADE, 120 were found within 90 different orthogroups, whereas 40 RGADE were not found forming orthogroups. The two sugarcane genotypes shared a total of 14 different orthogroups harboring all of the 61 RGADE predicted (Additional file 2).

**Fig. 4:**
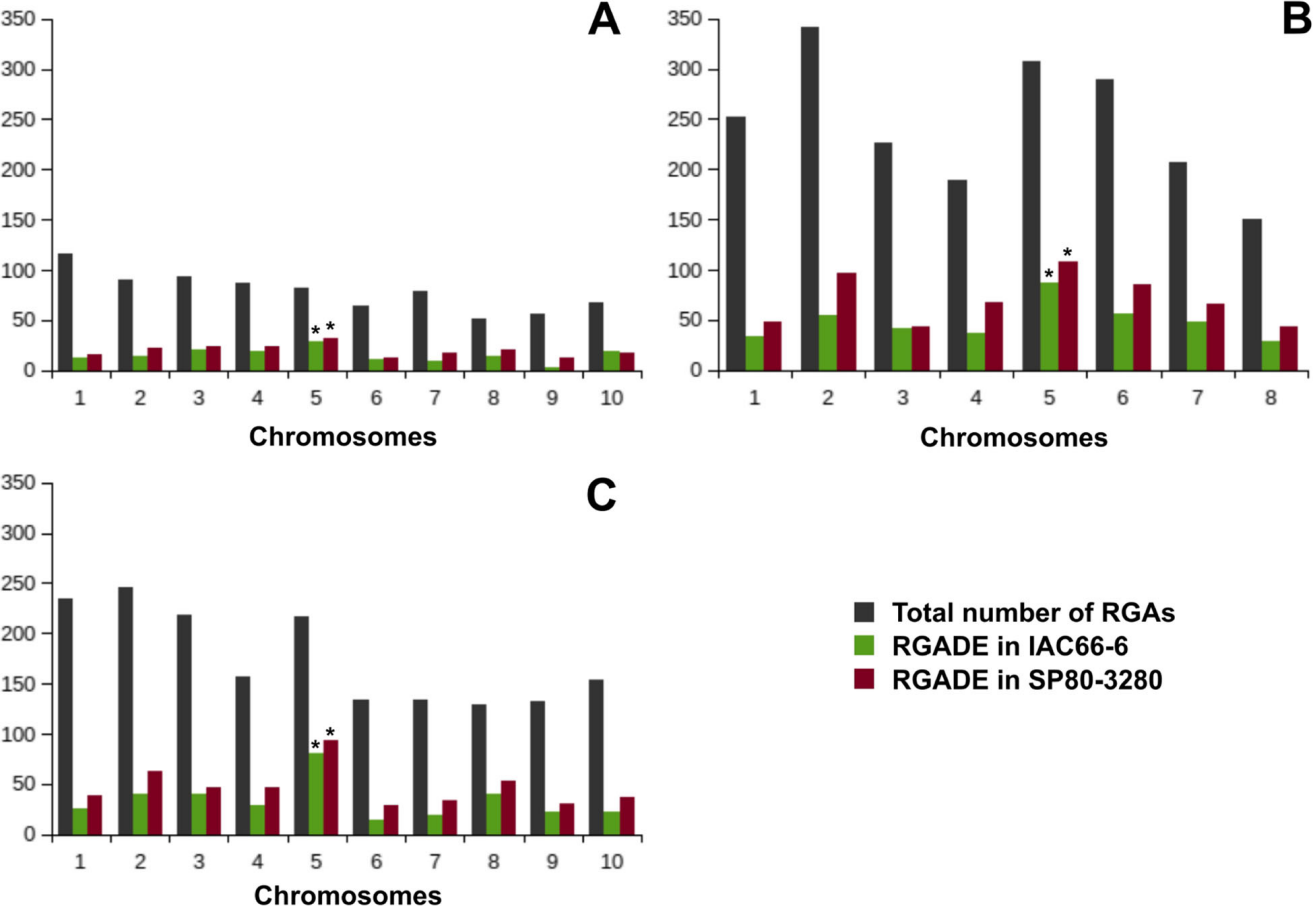
Overview of the chromosomal distribution of the total number of predicted RGAs, and the number of orthologs of RGADEs predicted within two sugarcane genotypes of IAC66–6 (smut-susceptible) and SP80–3280 (smut-resistant), in three genome references of sugarcane of: **a** R570, **b** AP85–441, and **c** *Sorghum bicolor*. Asterisks on the top of the bars indicate enrichment of RGADE orthologs at the level of *P* < 0.05 (Fisher’s exact test)

Although orthologs of RGADEs were distributed all along with the entire set of chromosomes of the three focal references, the proportion of RGADE orthologs in chromosome 5 was found increased in relation to the proportion of total RGAs predicted for this chromosome (Additional file 3: Table S2). In summary, the chromosome 5 was found enriched for orthologs of RGADEs, regardless of the genome reference used (Fig. 4; Additional file 2). Also, in general, there are more RGADEs responsive to smut in the resistant than in the susceptible genotype (Fig. 4).

Finally, we investigated whether the RGADE orthologs predicted within our three genome references were organized in clusters. The percentage of RGADE having orthologs organized in clusters comprised from 28 to 43% in relation to the total of predicted RGADE within each sugarcane genotype evaluated (Additional file 2). Orthologs from RGADEs predicted within the smut-susceptible sugarcane were 4% (in average) more frequently found within clusters as compared to the orthologs from smut-resistant RGADEs, regardless of which of the three genome references used for ortholog investigation (Additional file 2). Out of the 11 RGADE shared by the two sugarcane genotypes, 7 were found having orthologs organized in clusters in both the genomes of AP85–441 and sorghum, whereas 6 RGADE had orthologs organized in clusters in the genome of R570. In the AP85–441 genome, chromosomes Ss2 (*N* = 12) and Ss5 (*N* = 14) harbored the largest number of clusters having RGADE orthologs, whereas in the genome of R570, chromosomes Sh4 (*N* = 6) and Sh5 (*N* = 7) harbored them, and chromosomes Sb5 (*N* = 20) and Sb8 (*N* = 17) from sorghum.

### RGADEs evolutionary relationships

**Fig. 5:**
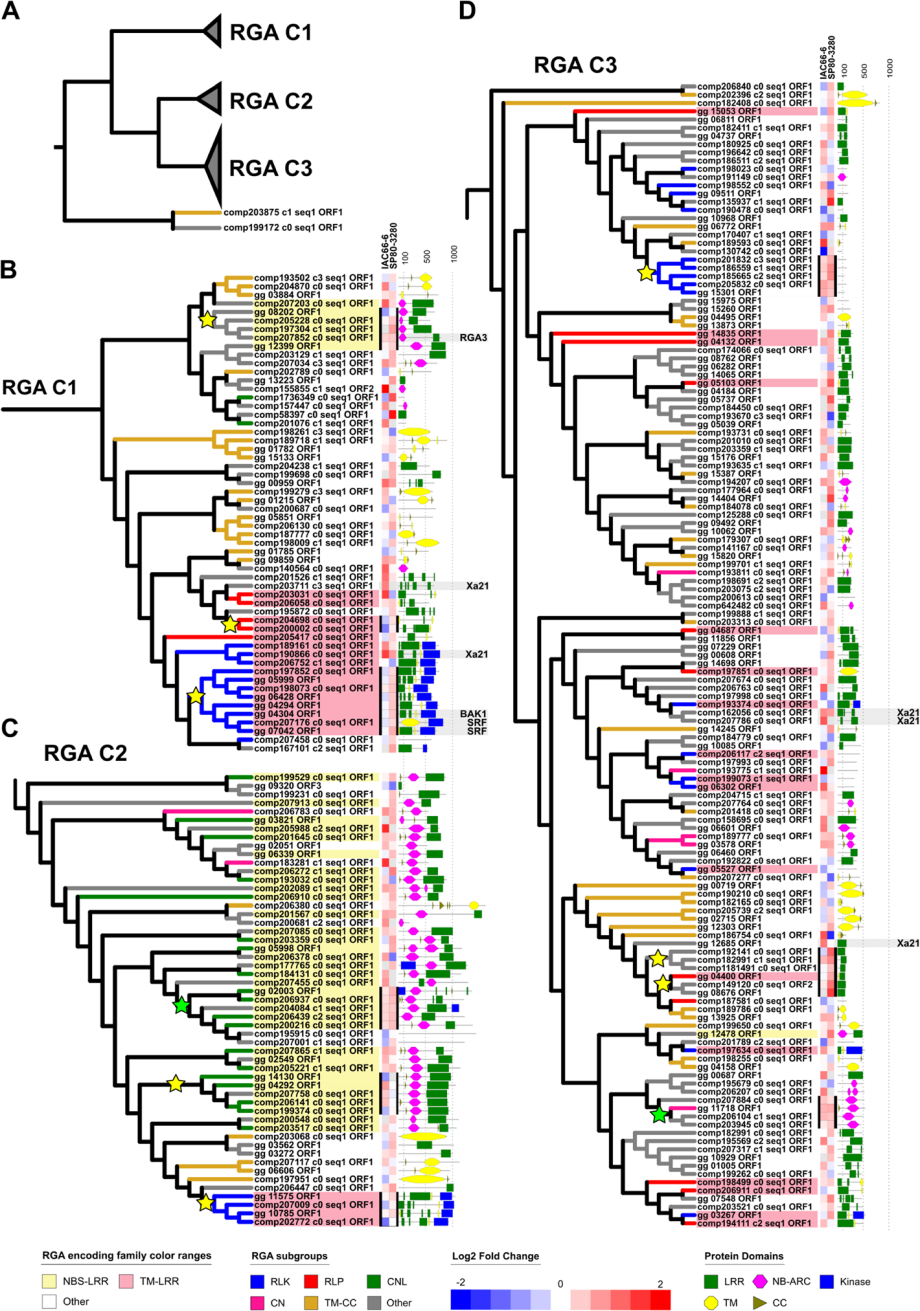
Phylogenetic tree showing the relationships among 250 RGADEs predicted within the COMPGG transcripts dataset. Cladogram was obtained with FastTree software and the Le-Gascuel model of amino acid evolution. **a** Full cladogram, with subclades collapsed. **b**, **c** and **d** Expanded RGA subclades. Expression profiles from sugarcane genotypes of IAC66–6 and SP80–3280 are represented as Log2 Fold Change values (inoculated/control) in the heatmap, if significant at *P <* 0.05 in at least one sugarcane genotype. In the heatmap, blue squares represent down-regulation, whereas red squares represent up-regulation; grey squares represent no transcript expression. The statistical significance of expression is fully presented in Additional file 2. Predicted protein domains are depicted with colors together with geometric forms accordingly to legend. RGAs not having depicted domains were predicted only based on orthology during BLASTp searches. Branches colors indicate RGA classes according to legend. Dashed grey lines indicate the amino acid sequence length. Green stars highlight clades where sugarcane genotypes shared related expression profiles. Yellow stars highlight clades where sugarcane genotypes had distinct expression profiles of RGADE

We investigated the evolutionary relationships among the predicted RGADEs through the use of maximum-likelihood phylogeny. The predicted RGA domains in addition to the RGAs expression profile (heatmaps) from both the two sugarcane genotypes (IAC66–6 and SP80–3280) were placed alongside to the obtained tree. The resulting tree (Fig. 5) split the predicted RGADEs into three main clades. RGAs from NBS-LRR encoding family were mainly grouped within the two clades of RGAC1 (Fig. 4B) and RGAC2 (Fig. 4C). One single NBS-LRR sequence was found within RGAC3 clade. Nested to RGAC1 clade, there were a few closely related TM-LRR subclades: three RLP subclades, and two RLK subclades. Another RLK subclade was nested to the clade RGAC2. No RLP sequences grouped within RGAC2 clade. Almost all NBS-LRR and TM-LRR RGA sequences found within RGAC1 and RGAC2 clades had domains predicted; few sequences were predicted through orthology. RGAC1 and RGAC2 clades also hosted TM-CC subclades, in addition to clades grouping other RGA variants. Most of these RGAs harboring variable domain combinations, and nested within RGAC1 and RGAC2 clades, formed single branches. TM-CC sequences formed subclades all across the tree, and showed close relationships to both NBS-LRR and TM-LRR encoding families. The RGAC3 clade (Fig. 4D) is sister to RGAC2, and hosted the largest number of RGADEs. Most of RGAs sequences within RGAC3 had domains predicted by our pipeline and a few were RLKs predicted through orthology searches.

The phylogenetic tree still depicted both the contrasting and related expression profiles of closely related RGAs between the two sugarcane genotypes. We highlighted nine subclades in Fig. 5 using green stars for subclades showing related RGA expression profiles among genotypes (*N* = 2), and yellow stars for subclades showing contrasting expression profiles of RGAs among genotypes (*N* = 8). The most striking finding came from TM-LRR subclades found within RGAC1 and RGAC2 clades. Two RLK subclades, and one RLP subclade comprised RGAs found as up-regulated in the smut-resistant SP80–3280 sugarcane genotype when compared to control plants. While there was an indication for down-regulation (blue squares) of these RGAs in the smut-susceptible IAC66–6 genotype, although the Log2 Fold Change values were not significant at *P* < 0.05.

We investigated to what extent the RGA orthologs from each of the three RGADE clades were also predicted as originated from tandem duplication events. Percentages of RGA orthologs tandem-derived were very similar among the three RGADE clades (Additional file 2). In the sorghum genome, RGADE orthologs were predicted as derived from tandem duplications between 53 to 58%. Percentages of tandem-derived RGADE orthologs were lower in the other two genome references: from 34 to 38% within R570 genome, and from 41 to 43% within the AP85–441 genome.

## Discussion

### RGA predictions in the sugarcane references

Here we used conserved structural features of both R and PRR genes to uncover RGAs within four references of sugarcane: 1) the monoploid genome of cultivar R570; 2) a monoploid version of the genome of *S. spontaneum* AP85–441; 3) the genome of sorghum; and 4) the COMPGG comprising a set of de novo unique transcripts generated from data of six modern sugarcane cultivars. Because RGAs may be encoded by a variety of domains/motifs combinations, we also used BLAST searches against the R genes reference database to predict RGA orthologs [2].

Among all the set of proteins sequences within each of the four focal sugarcane references, from 3.4% (in COMPGG) to 6.8% (in AP85–441) were predicted as RGAs (Table 1 These percentage results are higher compared to those found in literature for the ancient green plants such as *Physcomitrella patens* (1.6%) and *Selaginella moellendorffii* (1.3%), but are similar to those found for crops such as *Oryza sativa* (4%), and *Glycine max* (4.2%) [32].

Our predictions categorized RGAs into both the NBS-LRR and TM-LRR encoding families (Table 1). As previously reported by [33], we could not find RGAs from TNL class — TIR-NBS-LRR structure — in the sorghum genome. Likewise, the other three sugarcane references also lacked TNLs (Table 1). It is known that repeat masking approaches used to avoid the counting of transposon-related genes during genome annotations could impair the identification of TNL genes [34]. However, since we have also investigated sugarcane de novo transcriptome assemblies, impaired gene annotations may not be the cause for the lack of TNL within our targeted genome references. Furthermore, our findings reinforce other studies for the absence of TNL encoding genes in sugarcane [12] and other monocots [35–37].

Five were the most frequently observed classes of RGAs predicted within our four focal sugarcane references: CNL class from NBS-LRR encoding family, RLK and RLP classes from TM-LRR encoding family, in addition to TM-CC and CN classes. RGAs harboring other domains combinations were also predicted.

### Sugarcane RGA orthologs are organized in clusters and within conserved regions

We investigated the genomic features of the predicted RGAs specifically with regard to their chromosomal arrangement, cluster organization, and synteny. Our analysis showed that the RGAs are not evenly distributed across the chromosomes of the genome references, which agrees with a previous study that showed that 80% out of the 361 NBS-encoding genes identified in the genome of AP85–441 are located in four chromosomes: Ss2, Ss5, Ss6, and Ss7 [25]. We found a very similar pattern: of the 366 NBS-encoding genes predicted in AP85–441, 79% were located in the same chromosomes. Overall, 54% of the AP85–441 RGAs were found in syntenic segments. The largest syntenic segments harboring RGAs were found between the genomes of AP85–441 and sorghum (Fig. 2a), and between AP85–441 and R570 (Fig. 2b). Previous synteny analysis between AP85–441 and sorghum unveiled major chromosomal rearrangements in the *S. spontaneum* genome that reduced its number of chromosomes from 10 to 8. Inversions and rearrangements were predicted among chromosomes Ss2, Ss5, Ss6, and Ss7 of AP85–441 and Sb5 and Sb8 of sorghum [25]. In consonance with these previous findings [25], we encountered several syntenic segments (Fig. 2) among our three focal sugarcane genomic references, in addition to evidences of reorganization in the mentioned chromosomes. Major rearranged segments have been observed among Ss2, Ss5, Ss6, and Ss7 of AP85–441, and Sb5 and Sb8 of sorghum, with 51% of NBS-encoding genes identified to be located in those regions [25]. This result suggests that the non-rearranged chromosomal regions between AP85–441 and sorghum may represent conserved sources of disease resistance genes in these species. Thus, we investigated for similar retention patterns of NBS-encoding genes in chromosomes 2, 5, 6, 7, and 8 — all related to the rearrangements found in AP85–441 — in the genomes of sorghum and R570. The five chromosomes of sorghum harbored 72% of NBS-encoding genes, whereas the five chromosomes of R570 harbored 62% of NBS-encoding genes.

Differently from NBS-RGAs, the location of the TM-LRR ones were not biased towards either the set of four rearranged chromosomes of AP85–441 or in the five of sorghum and R570.

*S. spontaneum* is estimated to have contributed with 12.5% of the genomes of the modern sugarcane cultivars [25]. The RGA phylogenomic tree generated for 220 concatenated amino acid sequences of orthologous RGAs (Additional file 3: Figure S6d) suggests the genome of AP85–441 as having the closest evolutionary relationships with the clades of R570 and COMPGG. Therefore, the genome of *S. spontaneum* likely comprises an important source to understand disease resistance in modern sugarcane cultivars as previously proposed [25].

Another remarkable genomic organization feature of RGAs is their arrangement in clusters [3, 35]. Within the three focal genomic references, from 23 to 39% of the total predicted RGAs were found forming clusters. Further, cluster-arranged RGAs also presented high levels of shared synteny. About 40% of all the cluster-arranged RGAs were encountered within syntenic segments. The set of five chromosomes (2, 5, 6, 7, and 8) discussed above, interestingly harbored 57 and 59% of the total clusters of RGAs within the genomes of R570 and sorghum, respectively. The genome of AP85–441 had the largest percentage (61%) of predicted RGA clusters placed in the set of four chromosomes (Ss2, Ss5, Ss6, and Ss7).

We also investigated if tandem duplications have contributed to the expansion of predicted clusters of RGAs. Tandem duplications have been attributed to contribute greatly for RGA evolution and the rise of novel specificity [38]. Our analysis supports tandem duplication events as the origin of about 46% of the cluster-arranged RGAs within the R570 genome. In the other two genomes of AP85–441 and sorghum, 48 and 62%, respectively, of the cluster-arranged RGAs were also predicted as tandemly duplicated.

### Smut-resistant sugarcane has increased differential expression of innate immune system

We investigated the RGA transcriptome profiles of two sugarcane genotypes with contrasting degrees of resistance to smut at the early stage of interaction (48 hai) with *S. scitamineum*. The susceptible genotype (IAC66–6) had a higher number of overall expressed genes compared to the resistant one (SP80–3280). However, both the number of DEGs and the number of RGADEs were higher in the smut-resistant sugarcane. Differences in transcriptome profiles among sugarcane varieties during interaction with *S. scitamineum* have been previously reported [21, 39, 40] but in this study we focused on the analysis of RGADE profiles.

The two focal sugarcane genotypes presented a very distinct profile of RGADEs. Only 11 RGADEs were shared between the two genotypes. Disparities among the two sugarcane RGADE profiles were observed in the two layers of the plant immune system. With respect to the first layer, comprised by the transmembrane leucine-rich repeat (TM-LRR) encoding family proteins, the targeted smut-resistant sugarcane (SP80–3280) had a larger number (*N* = 18) of significantly up-regulated RLK as compared to the number (*N* = 6) of predicted up-regulated RLK in the smut-susceptible sugarcane (IAC66–6). In addition to the quantitative divergence of RLK expression between genotypes, the smut-resistant SP80–3280 also had two exclusively up-regulated RLKs (comp207176_c0_seq1, gg_07042). Orthologs of them have been previously reported to interact and positively regulate plant immunity: 1) a LRR-RLK BRI1-Associated Receptor Kinase1 Bak1; and 2) a leucine-rich repeat transmembrane receptor-like kinase Strubbelig-Receptor Family (SRF). Bak1 has been shown as essential to trigger resistance to various pathogens through the production of reactive oxygen species [41]. The increased transcription of SRF genes was recently demonstrated to respond to environmental stimuli [42]. Furthermore, the StLRPK1 gene product from SRF was demonstrated to interact with Bak1 to mediate potato immunity against *Phytophthora infestans* [42]. Bak1 has also been previously related to smut in both transcriptomic and proteomic data [21, 43].

Genotypes shared one single RLK up-regulated (comp190866_c0_seq1), which was annotated by the Blast2GO software as an ortholog of the receptor kinase-like protein *Xa21* of rice. *Xa21* is known to promote innate immunity by detecting *Xanthomonas oryzae* pv. *oryzae* protein Ax21 [44]. The *Xa21* (comp190866_c0_seq1) was found by the OrthoMCL within a large a group of orthologs GRU2 (*N* = 245), grouping sequences from all the four focal references of this study. Regarding the second layer of the plant immune system, consisted by the NBS-LRR encoding family proteins, sugarcane presented only RGAs from the class of CNL (CC-NBS-LRR). These were differentially expressed upon inoculation with *S. scitamineum*. Similarly, to the pattern we have found for the RGAs from the first layer, the expression of the CNL RGAs differed between the resistant and susceptible genotypes. One single CNL (comp207865_c1_seq1) annotated as a putative disease resistance protein RGA3 was up-regulated in both genotypes. This sequence belongs to the ortholog group GRU18390 which is consisted of only two sequences, among the COMPGG and AP85–441 references. *We also investigated for orthologs of the* brown rust resistance gene *Bru1* first identified in the R570 genotype [45]. *Bru1* has proven to be a major dominant resistance gene in sugarcane and has its origin previously identified as from *S. spontaneum* [46, 47]. *Bru1* is suggested to encode an ortholog of the serine (S)/threonine (T) kinase *Rpg1* and lays on a cluster of other S/T kinase [46]. In our analysis, the ortholog *Rpp1*-like/*Bru1* (comp207914_c0_seq1) was not responsive to smut.

At the level of ortholog groups, we could find more functional relationships among the RGADE profiles from the two sugarcane genotypes. Smut-resistant and smut-susceptible genotypes shared 14 orthogroups. Within each of the 14 orthogroups, RGADEs presented elevated sequence similarity and are likely related in function. In total, were 61 RGADE composing the shared orthogroups. Therefore, 38% (*N* = 39) of the total of RGADE predicted within the smut-resistance sugarcane transcriptome is related in both sequence and function to 20% (*N* = 32) of the total of RGADEs predicted within the smut-susceptible sugarcane genotypes. Alternatively, it has been demonstrated that approximately 14% of the genes from sugarcane modern variety RB925345 are alternatively spliced during infection with smut (Bedre et al., 2019). Each orthogroup containing RGADEs and predicted as shared by the genotypes were composed exclusively by any of the: TM-LRR (*N* = 1 orthogroup); and NBS-LRR (*N* = 3 orthogroups) encoding families; or TM-CC class (*N* = 2 orthogroups). In addition, orthogroups also contained RGADEs harboring other domains combination. Finally, TM-LRR may comprise major disparities among RGADE profiles of the two investigated sugarcane genotypes since a unique orthogroup from aforementioned encoding family, containing only 6 RGADE in total, was predicted as shared among the genotypes. TM-LRRs are pattern recognition receptors (PRR) comprising the innate plant immune system and are able to recognize directly from cell surface a wide range of PAMPs and promote PAMP-triggered immunity (PTI). No ETI has been yet identified for the smut-sugarcane pathosystem. On the other hand, oxidative burst was described for the early stages (5 days after inoculation) of sugarcane interaction with smut [28]. Therefore, divergences among RGADE profile involving TM-LRR may be associated to the smut-resistance observed for the sugarcane variety SP80–3280, in addition to the increased number of RGADEs from both classes of RLK and RLP as compared to the smut-susceptible genotype.

We did not detect the presence of a major resistance gene influencing resistance to smut as for other pathosystems [48]. However, attention maybe given in future studies to orthologs of Bak1. Instead, a combination of various RGAs mostly of the TM-LRR class responded to the pathogen infection in resistant plants 48 hai. These various RGAs were detected as having orthologs enriched in chromosome 5 of the ancestral genotype. The same pattern of distribution was not detected in the modern cultivars in this work.

### RGA families have divergent expression profiles between sugarcane genotypes

In the phylogeny of RGADEs, the NBS-LRR and TM-LRR encoding families were closely related. The two major clades of RGAC1 and RGAC2 grouped mostly of the RGADE sequences harboring well-defined domains. In addition, most of RGADEs predicted from the two aforementioned major encoding families were grouped in the RGA clades RGAC1 and RGAC2. Clade RGAC3 was closely related to RGAC2. Conversely to the two other clades, the RGAC3 grouped sequences that either presented more variable combination of domains or were predicted as RGA exclusively during orthology predictions.

Contrasting RGADE profiles between the two focal sugarcane genotypes were observed for most of the clades composed by TM-LRR sequences. These data suggest that divergences observed between the two focal sugarcane profiles of RGADE associated with the first layer of sugarcane immune system are indeed related to function — as suggested throughout RGADE orthogroups comparison —, rather than only the number of RGADE. For instance, the RGAC1 grouped most of the TM-LRR up-regulated genes in the resistant genotype, which included orthologs of Bak1 (Fig. 5). As mentioned before, Bak1 is known to be involved in various signaling pathways, including those associated with resistance to pathogens and herbivores [41, 42, 49].

Expansion of the predicted RGADE clades is a result of tandem duplications. Within clades, RGADE orthologs were up to 58% found as derived from tandem duplications. Percentages of RGADE orthologs derived from tandem duplications were very similar among the three major clades predicted. Tandem duplicates are believed as having higher turnover rates as compared to genes duplicated by larger duplication events such as WGD [50]. Accordingly, RGAs have been thought as fast-evolving genes, with the mechanisms of unequal crossing-over, recombination, gene conversion, transposition, and gene duplication producing variability, and giving rise to subfamilies [51, 52]. Novel resistant phenotypes have also been attributed to events of reorganization and evolution of resistance genes [53].

## Conclusions

In summary, our findings showed sugarcane references as composing a set of 7703 RGAs distributed in 1459 ortholog groups. The most abundant class of RGA identified were those of TM-CC. Sugarcane did not present class TNL of RGAs. Chromosomes 02, 05, 06, 07, and 08 were the ones harboring the highest number of RGA clusters and RGAs derived from tandem duplications. Chromosome 5 in the ancestral genotype (*S. spontaneum*) is potentially the origin of most RGAs responsive to smut in modern sugarcane varieties. Smut resistant and susceptible genotypes of sugarcane have a distinct pattern of RGAs expression, probably related to their genealogy, allele composition, and eventually alternative splicing that we did not consider in our analysis. The TM-LRR encoding family was the most responsive to the pathogen infection (up-regulated) in the resistant genotype in the early moments of the interaction, suggesting the relevance of an innate immune system as the first response. Specifically, the resistant genotype had an increased number of RGAs differentially expressed from both classes of RLK and RLP as compared to the smut-susceptible genotype. Phylogenetic studies defined three main RGADE clades RGAC1–3. RGAC1 grouped most of the TM-LRR up-regulated in the resistant genotype, including orthologs of Bak1. We believe that this work can help to outline strategies for further understanding of allele and paralog expression of RGAs in sugarcane, and the results should help to develop of a more applied procedure for the selection of resistant plants in sugarcane.

## Methods

### Plant material, RNA extraction, libraries, and sequencing

Three biological replicates of two sugarcane genotypes with different degrees of resistance to smut were used in this study (Additional file 3: Figure S7). First, single-bud sets of 10-month-old healthy plants of the IAC66–6 (smut-susceptible genotype) and SP80–3280 (resistant genotype) were inoculated using SSC39 teliospores following as previously described by [54]. The sugarcane genotypes used in this work have different genealogy: the IAC66–6 is derived from the cross between Co419 x Co350, and has a recent ancestral in *Sorghum durum*; the SP80–3280 is derived from the cross between SP71–1088 x H57–5028 (IAC Sugarcane breeding Program databank - Caiana). The healthy buds used to conduct the experiments were obtained from IAC sugarcane nursery. No special permits were necessary for teliospores and genotypes used, because this project was developed in collaboration with IAC researchers. This work does not involve endangered or protected species. Twenty buds of each genotype were collected at each of the time point of 6, 12, 24, 48 and 72 h post-inoculation (hpi) [28]. The plant material collected at the time point of 48 hpi was chosen for the development of this study.

Total RNA was extracted from the samples using distinct methods for each plant developmental stage as described by Taniguti et al. (2015). The quality of the total RNA was verified using an Agilent 2100 Bioanalyzer (Agilent Technologies, USA), and the libraries were constructed using a TruSeq RNA Sample Prep v2 Low Throughput (LT) kit as described in the manufacturer’s instructions (Illumina, San Diego, CA). The libraries were paired-end sequenced using the Illumina system (HiScanSQ).

### Genomic and transcriptomic data collection

Protein sequences and genomic annotation were obtained for three sugarcane references: 1) for the monoploid genome of cultivar R570 [24], data were downloaded from http://sugarcane-genome.cirad.fr/; 2) data for a monoploid version of the allele defined genome of *S. spontaneum* AP85–441 [25] was kindly provided by the authors; and 3) *S. bicolor* data was downloaded from PLAZA monocots 4.0 [29]. Additionally, a set of 72,269 unique de novo transcripts from six sugarcane genotypes [30] was obtained alongside with 16,219 de novo assembled transcript sequences from variety RB925345 [21], to assembly the COMPGG sugarcane transcript reference dataset (*N* = 88,488). Finally, 152 reference R genes protein sequences were downloaded from Pathogen Receptor Genes database (PRGdb) [31].

### RGA predictions by structural analysis

We investigated RGAs amongst the protein sequences from each of the three sugarcane references, in addition to the sorghum protein sequences used in this study, based on R genes conserved features. Thus, we used five softwares to predict conserved domains/motifs of R genes: 1) InterProScan v5.33–72.0 [55] with the analyses of Coils-2.2.1, Gene3D-4.2.0, Pfam v32.0, SMART-7.1, and SUPERFAMILY-1.75; 2) PfamScan with Pfam v32.0 [56]; 3) a standalone version of Phobius [57]; 4) TMHMM v2 [58]; and 5) Coils v2 [59]. Each software searched for specific or multiple R genes conserved features of: Leucine-rich repeats (LRR), Protein kinase domains, Serine-threonine/tyrosine kinase (STTK), Lysine motifs (LysM), Toll/interleukin-1 receptor (TIR), Coiled-coil (CC), and Nucleotide-Binding associated to ARC (NB-ARC), Nucleotide-binding associated to LRR (NB-LRR), and Transmembrane (TM). Custom Python3 scripts were then used to parse each of the softwares outputs and classify RGA candidates if harboring a set of domains/motifs accordingly: 1) TM-LRR encoding family: RLK (TM + LRR or NB-LRR + kinase domains), RLP (TM + LRR or NB-LRR or LysM); 2) NBS-LRR encoding family: TN (TIR + NBS/NB/NB-ARC), TNL (TIR + NB-ARC + LRR or NB-LRR), CN (CC + NB-ARC), CNL (CC + NB-ARC + LRR or NB-LRR); 3) Other domains combinations: TM-CC (TM + CC), TIR (TIR), Other variants. Only sequences harboring at least one out of three RGA basic domains — LRR, NB-ARC, or NB-LRR — were kept to assembly the RGA candidates databases for each of the three references (Additional file 2; http://amos.esalq.usp.br/sord/). We applied our pipeline to a set of R reference genes from PRGdb for validation. Out of 152 genes, we excluded 24 genes lacking domain information (Additional file 1). Only 128 R genes were retained in the PRGdb dataset for the validation step of predicting RGA candidates and downstream analysis.

### RGA prediction by orthology searches (BLASTp)

R genes may be encoded in a variety of combinations [8], and may require the formation of multi-protein R-complexes to trigger signaling [51]. For example, the R gene *Pto* is comprised of only a protein kinase domain and requires association to the NBS–LRR gene *Prf* for function. Accordingly, to the structural features expected for detecting RGAs implemented in our pipeline, the prediction of a solely kinase domain would not classify a sequence such as the *Pto* as RGA. Thus, we used an additional analysis to find putative R orthologs and supplement each of the RGA candidates databases. Protein sequences from each sugarcane references were used as queries during BLASTp searches against sequences from PRGdb. Queries having an e-value <1e-05, minimum of 40% of identity, and query coverage percentage > 85% were added accordingly to each of the four references RGA candidates databases (Additional file 2).

### Sugarcane RGA orthologous relationships

We used the Markov Cluster algorithm implemented in the OrthoMCL v2.0.9 software [60] to establish orthologous relationships among the total set of RGAs from the four focal sugarcane references. During BLASTp all-vs-all step, we used the total set of protein sequences from the four focal sugarcane references of this study as both query and database, with an e-value cutoff of e^− 05^. OrthoMCL software generates clusters of proteins consisting of orthologs from at least two species. The clusters predicted by the OrthoMCL were then assumed as ortholog groups (orthogroups).

### Sugarcane RGA clusters and shared synteny analysis

We used homemade Python3 scripts to predict RGA clusters within the four sugarcane references using a method adapted from [3]. Clusters were established among at least 3 putative RGA, from any of predicted classes, if: 1) between two neighboring RGAs there were no more than 9 other genes; and 2) two neighboring RGAs were not separated apart by more than 250 kb.

Shared synteny among the predicted RGAs within the three targeted sugarcane genome references was also investigated. Firstly, we performed a BLASTp all-against-all searches with an e-value cutoff of e^− 05^. Custom Python3 scripts were used to parse the BLASTp tabular output in order to prepare input files for the DAGchainer software [61]. DAGchainer was run ignoring tandem duplication alignments, with 250,000 bp set as maximum distance allowed between two matches, and only segmental regions of at least six gene pairs were kept. For both the cluster investigation and synteny analysis, only the RGAs assigned to chromosomes were used. Figures were prepared using Circos [62] and the R package ggplot2.

### Tandem-derived RGAs

The RGA content of the three genomic sugarcane references were investigated to assess whether tandem duplications were responsible for their origin. A custom Python3 script was used to parse BLASTp all-against-all searches and keep only non-self matches within each of the three references. An accessory segmental duplication tool made available alongside DAGchainer was used to find collinear sets of homologous genes, with the ‘max intervening genes value’ set to 10.

### Reference-based transcriptome assembly

Raw Illumina paired-end reads were firstly preprocessed. Reads quality were checked using the FastQC v0.11.5 software (http://www.bioinformatics.babraham.ac.uk/projects/fastqc/). Adaptors were filtered out with Cutadapt v1.18 [63], still delivering only reads with no N bases, length > 20 bp, and average Q > 20. Pre-processed paired-end reads were mapped against a reference set of COMPGG sugarcane transcripts using the HISAT2 v2.1.0 software with default parameters [64].

### Transcript expression analysis

Counting tables were obtained parsing the mapping BAM files to the FeatureCounts software, from Subread package [65]. The EdgeR Bioconductor software package [66] was then used to identify the Differentially Expressed Genes (DEGs). DEGs were considered as statistically significant if *P* < 0.05, and were represented as values of a Log2 Fold Change (inoculated/control).

### RGAs phylogenetic relationships

We used maximum-likelihood phylogeny to investigate the relationships among predicted RGADEs. First, RGADEs protein sequences were aligned using Muscle [67] with the parameters set for the fastest possible alignment for amino acids. The phylogeny was inferred using the FastTree v2.1.10 SSE3 software [68] with the LG model of amino acid evolution [69], in addition to parameters -bionj and -slow. Final cladogram was obtained and visualized using iTOL v4.3.2 [70].

## Supplementary information

**Additional file 1.** PRGdb Dataset.

**Additional file 2.** RGA Dataset.

**Additional file 3: Figure S1.** Distribution of RGA candidates predicted from COMPGG de novo unique transcript sequences along 10 chromosomes of *Sorghum bicolor* genome. **Figure S2.** Distribution of RGA candidates predicted from AP85–441 *S. spontaneum* along 10 chromosomes of *Sorghum bicolor* genome. **Figure S3.** Distribution of RGA candidates predicted from R570 sugarcane cultivar monoploid genome along 10 chromosomes of *Sorghum bicolor* genome. **Figure S4.** Distribution of RGA candidates predicted from *Sorghum bicolor* along 10 chromosomes of its genome. **Figure S5.** Shared synteny view of the four most frequent RGA subgroups along the chromosomes of three references of sugarcane. **Figure S6.** RGA ortholog relationships among four references of sugarcane. **Figure S7.** Experimental design used in this work. **Table S1.** General RNAseq data statistics used in this study. **Table S2.** Overview of the proportion of predicted RGAs and RGADEs in the chromosomes in relation to the total of correspondent predictions within each of three sugarcane genome references.

## Data Availability

Raw transcriptomic data generated in this study have been submitted to the NCBI BioProject database (http://www.ncbi.nlm.nih.gov/bioproject/) under accession number PRJNA546134. BioSample accessions: SAMN11953770, SAMN11953771, SAMN11953772, SAMN11953773. The RGA dataset prepared for this study (Additional file 2) is also available online at the Sugarcane Orthologs of Resistance Database (SORD) (http://amos.esalq.usp.br/sord). Code for the reproduction of the analyses within this paper are available on GitHub (http://github.com/hugorody/rga).

## Abbreviations

BAC: Bacterial Artificial Chromosomes
CC: Coiled Coil domain
CN: CC domain associated to NB-ARC
CNL: CC domain associated to NB-ARC followed by LRR
DEG: Differentially Expressed Genes
EST: Expressed sequence tag
ETI: Effector-Triggered Immunity
hpi: hours post-inoculation
LRR: Leucine-rich Repeat
LysM: Lysine motif
NB: Nucleotide Binding
NB-ARC: Nucleotide Binding associated to ARC
NB-LRR: Nucleotide-binding associated to LRR
NBS: Nucleotide Binding Site
NBS-LRR: NBS/NB-ARC domain followed by LRR
PAMP: Pathogen-associated molecular patterns
PRGdb: Plant Resistance Genes database
PRR: Pattern Recognition Receptors
PTI: PAMP-Triggered Immunity
R: Resistance gene
RGA: Resistance Gene Analog
RGADE: Resistance Gene Analog Differentially Expressed
RLK: Receptor-like kinase
RLP: Receptor-like protein
SRF: Strubbelig-Receptor Family
STTK: Serine-threonine/tyrosine kinase
TIR: Toll/interleukin-1 receptor
TM: transmembrane domains
TM-CC: Transmembrane domain associated to CC
TM-LRR: TM followed by LRR
TN: TIR domain associated to NB-ARC
TNL: TIR domain associated to NB-ARC and LRR
WGD: Whole-genome duplications
