# Supplementary material for: Genome survey of resistance gene analogs in sugarcane: genomic features and differential expression of the innate immune system from a smut-resistant genotype
Source: BMC Genomics. 2019 Nov 6;20:809. doi: 10.1186/s12864-019-6207-y (PMC6836459; doi:10.1186/s12864-019-6207-y)
Supplement: Supplementary file 3 — Additional file 3: Figure S1. Distribution of RGA candidates predicted from COMPGG de novo unique transcript sequences along 10 chromosomes of Sorghum bicolor genome. Figure S2. Distribution of RGA candidates predicted from AP85–441 S. spontaneum along 10 chromosomes of Sorghum bicolor genome. Figure S3. Distribution of RGA candidates predicted from R570 sugarcane cultivar monoploid genome along 10 chromosomes of Sorghum bicolor genome. Figure S4. Distribution of RGA candidates predicted from Sorghum bicolor along 10 chromosomes of its genome. Figure S5. Shared synteny view of the four most frequent RGA subgroups along the chromosomes of three references of sugarcane. Figure S6. RGA ortholog relationships among four references of sugarcane. Figure S7. Experimental design used in this work. Table S1. General RNAseq data statistics used in this study. Table S2. Overview of the proportion of predicted RGAs and RGADEs in the chromosomes in relation to the total of correspondent predictions within each of three sugarcane genome references. [file 12864_2019_6207_MOESM3_ESM.pdf]

Additional file 3
Supplementary Data

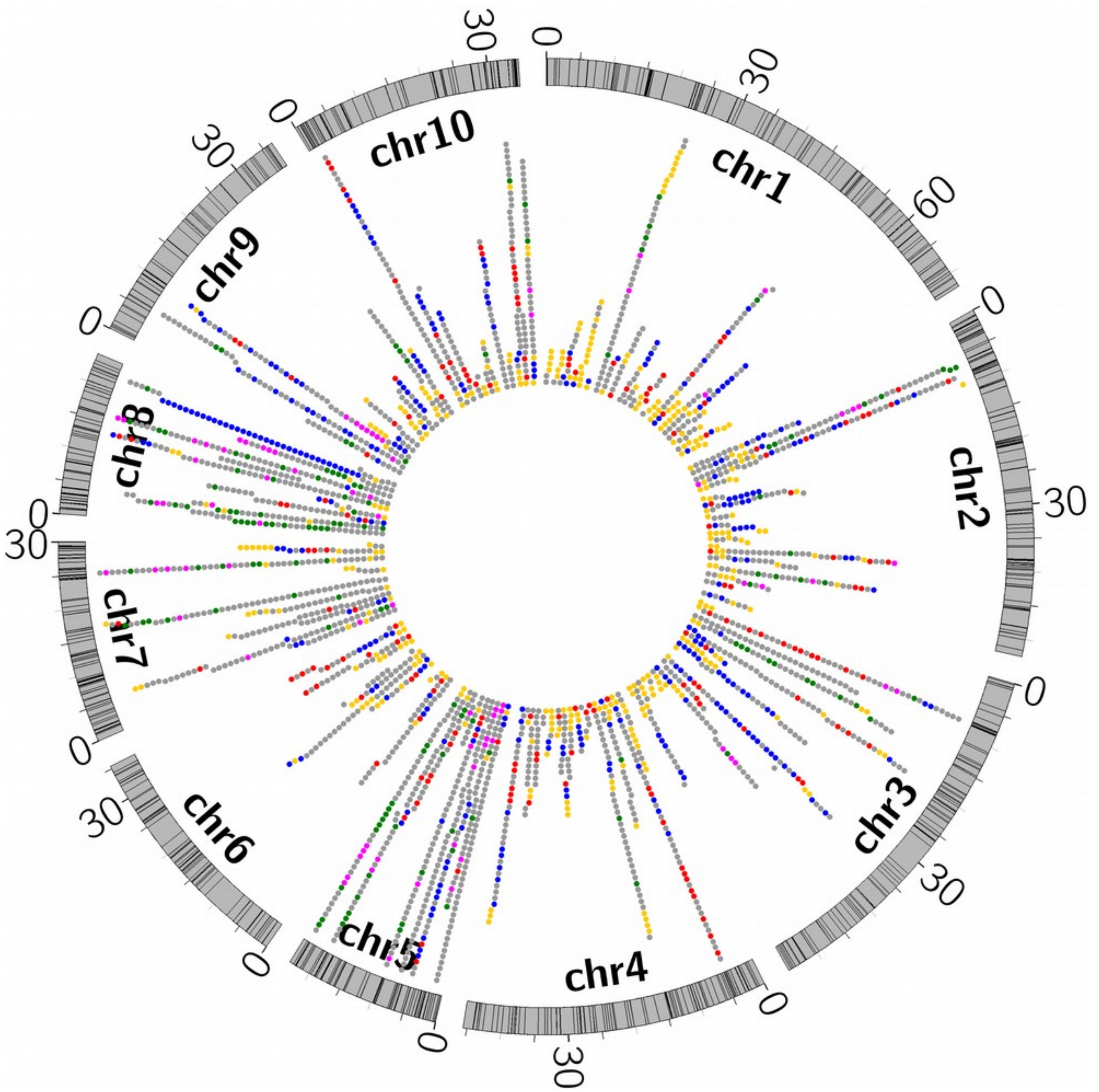

Supplementary Figure S1. Distribution of RGA candidates predicted from COMPGG *de novo* unique transcript sequences along 10 chromosomes of *Sorghum bicolor* genome. The ring indicate the chromosomes in Mbp. Traces in chromosomes indicate RGAs positions. Colored dots indicate RGAs accordingly to subgroup: RLK: blue; RLP: red; CNL: green; TM-CC: yellow; CN: purple; Other variants: grey. Figure Swas prepared using the software Circos.

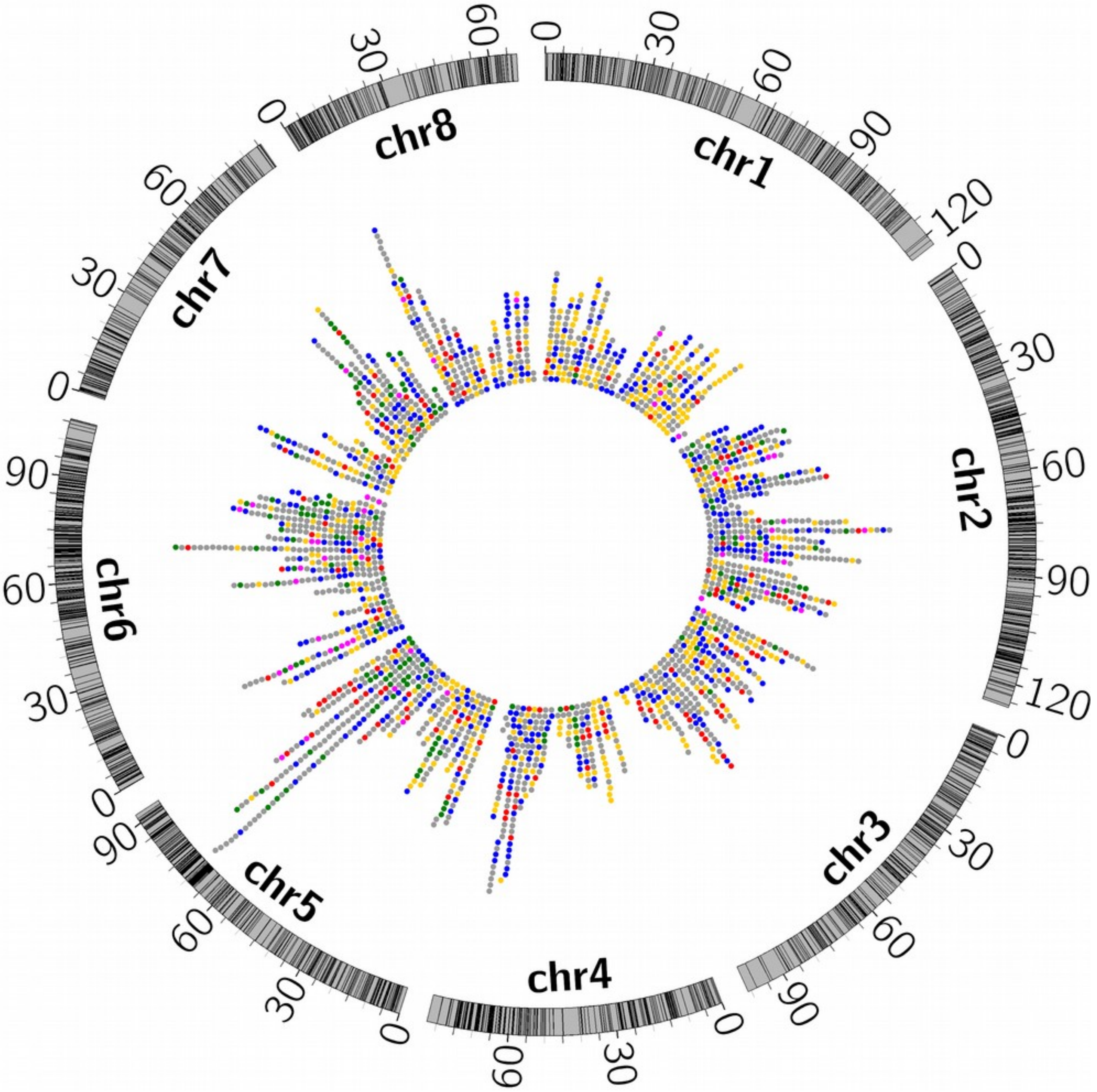

Supplementary Figure S2. Distribution of RGA candidates predicted from AP85-441 *S. spontaneum* along 10 chromosomes of *Sorghum bicolor* genome. The ring indicate the chromosomes in Mbp. Traces in chromosomes indicate RGAs positions. Colored dots indicate RGAs accordingly to subgroup: RLK: blue; RLP: red; CNL: green; TM-CC: yellow; CN: purple; Other variants: grey. Figure Swas prepared using the software Circos.

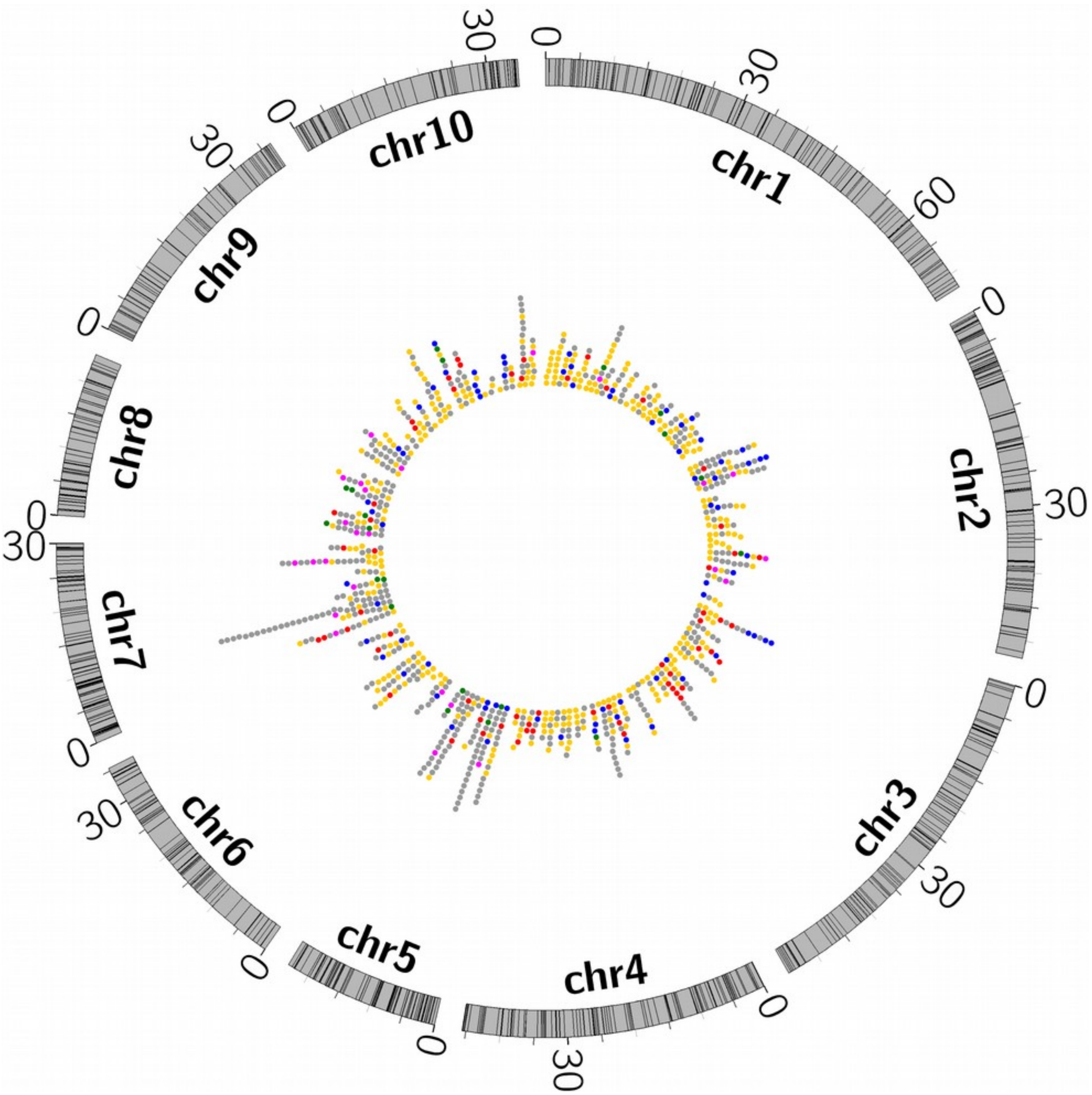

Supplementary Figure S3. Distribution of RGA candidates predicted from R570 sugarcane cultivar monoploid genome along 10 chromosomes of *Sorghum bicolor* genome. The ring indicate the chromosomes in Mbp. Traces in chromosomes indicate RGAs positions. Colored dots indicate RGAs accordingly to subgroup: RLK: blue; RLP: red; CNL: green; TM-CC: yellow; CN: purple; Other variants: grey. Figure Swas prepared using the software Circos.

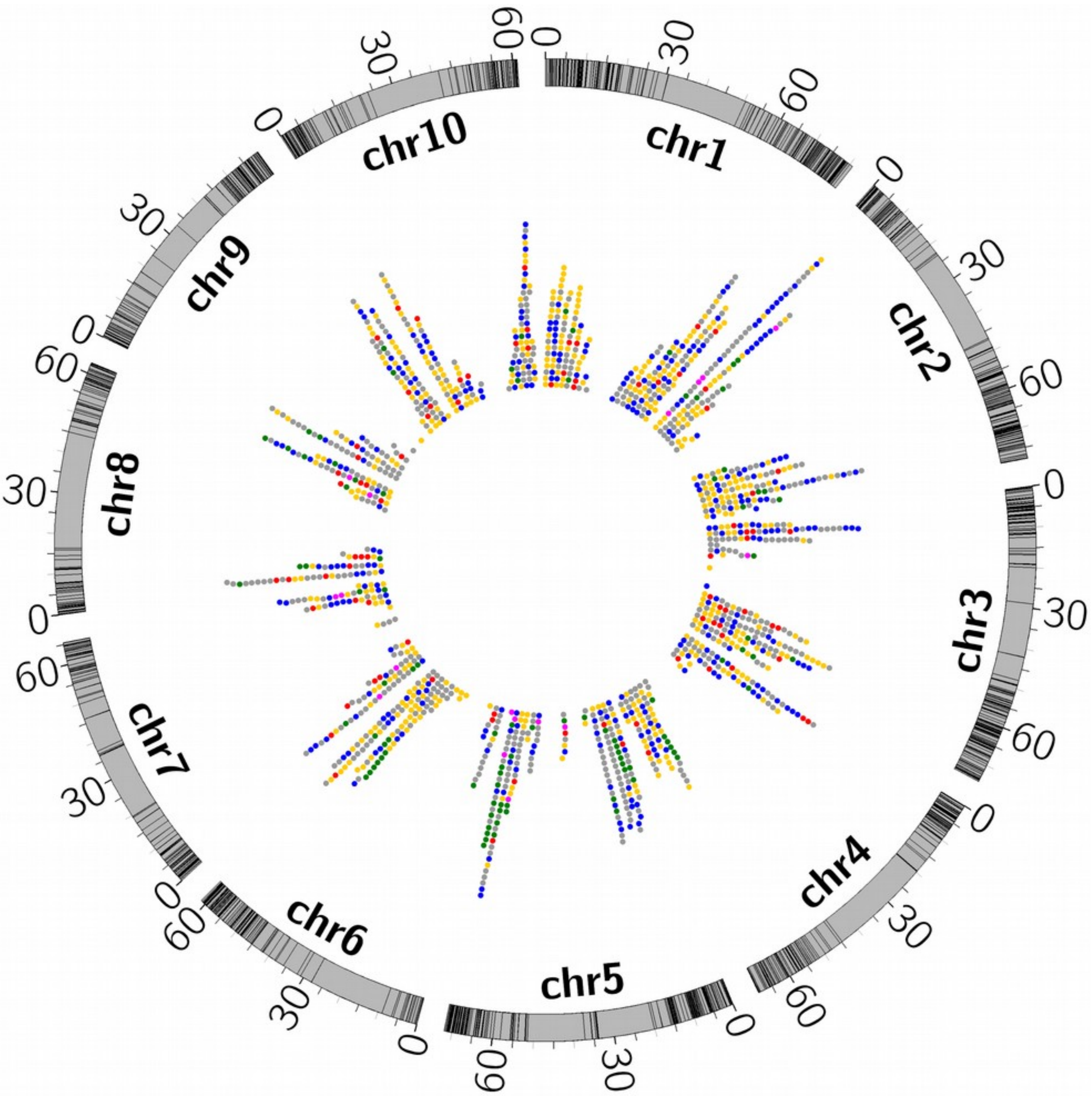

Supplementary Figure S4. Distribution of RGA candidates predicted from *Sorghum bicolor* along 10 chromosomes of its genome. The ring indicate the chromosomes in Mbp. Traces in chromosomes indicate RGAs positions. Colored dots indicate RGAs accordingly to subgroup: RLK: blue; RLP: red; CNL: green; TM-CC: yellow; CN: purple; Other variants: grey. Figure Swas prepared using the software Circos.

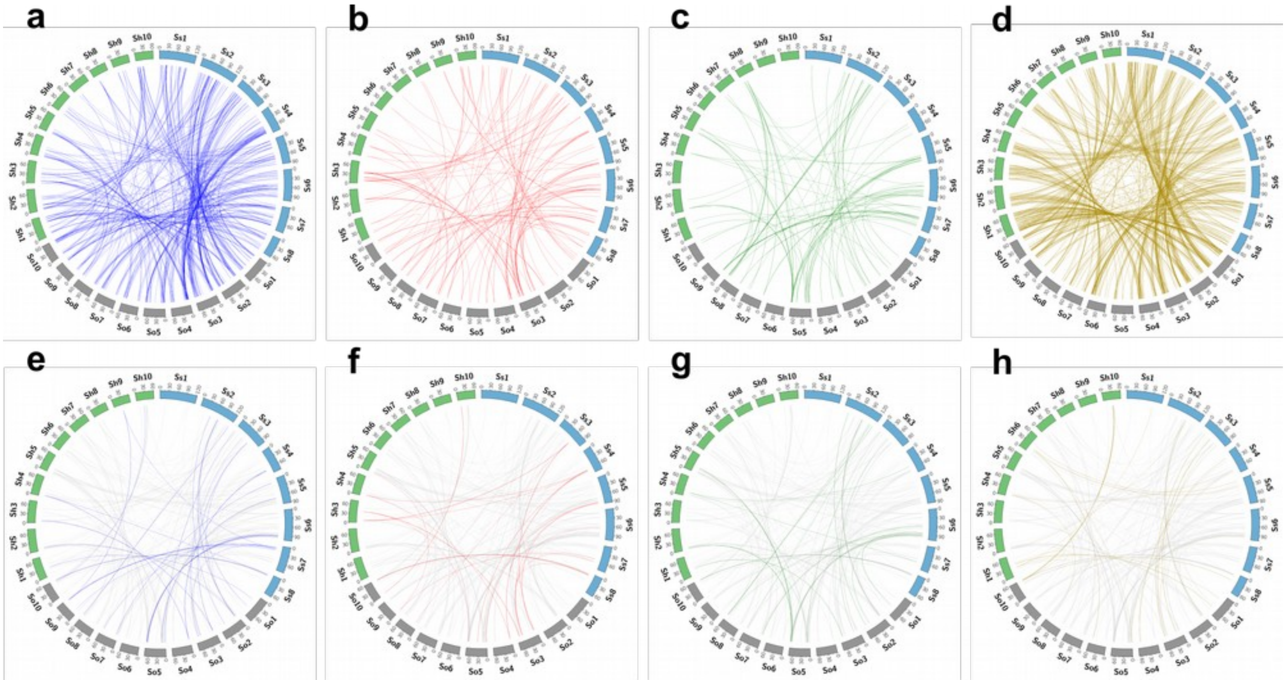

Supplementary Figure S5. Shared synteny view of the four most frequent RGA subgroups along the chromosomes of three references of sugarcane. All predicted RGAs in subgroups: **a** RLK. **b** RLP. **c** CNL. **d** TM-CC. All predicted RGAs arranged in clusters with subgroups highlighted in: **e** RLK. **f** RLP. **g** CNL. **h** TM-CC. Links represents reciprocal best-hit genes among references obtained by BLASTp searches (e-value < $1e^{-20}$, identity > 40, ranked by query coverage). Outer circle represents the chromosomes, with scale showing coordinates in base pairs, and colored accordingly: AP85-441: blue; *S. bicolor*: grey; and R570: green.

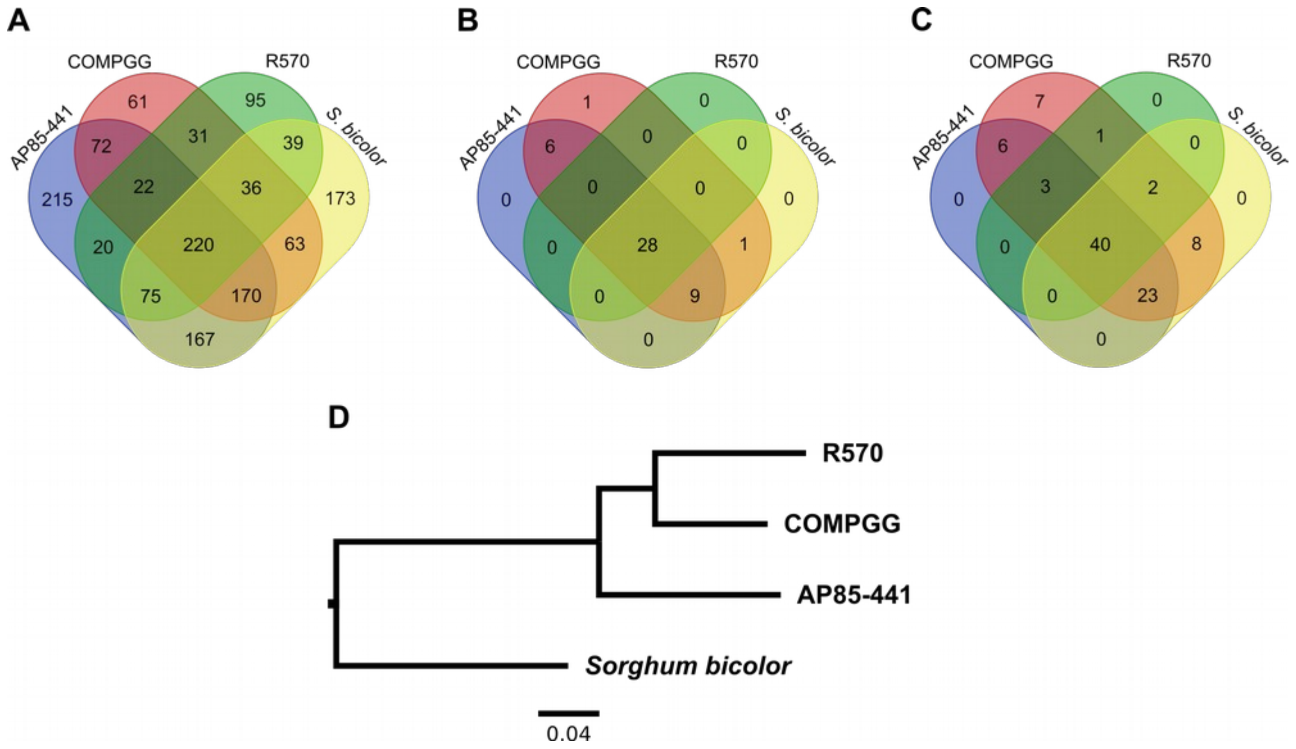

Supplementary Figure S6. RGA ortholog relationships among four references of sugarcane. Venn diagrams showing the number of orthogroups harboring: **A**, RGAs among the four focal references for sugarcane; **B**, RGADE predicted within sugarcane IAC66-6 cultivar; and **C**, RGADE predicted within sugarcane SP80-3280 cultivar. Orthogroups were predicted using OrthoMCL with BLASTp cutoff e^{-05}. Figure Swas produced with Draw Venn Diagram web application (http://bioinformatics.psb.ugent.be/webtools/Venn/). **D**, Phylogenetic tree showing the relationships among four references of sugarcane obtained from 220 concatenated RGA loci found shared among the four focal sugarcane references used in this study. Tree was obtained having the multiple-alignment of protein sequences performed by Muscle as input to FastTree software and the Le-Gascuel model of amino acid evolution.

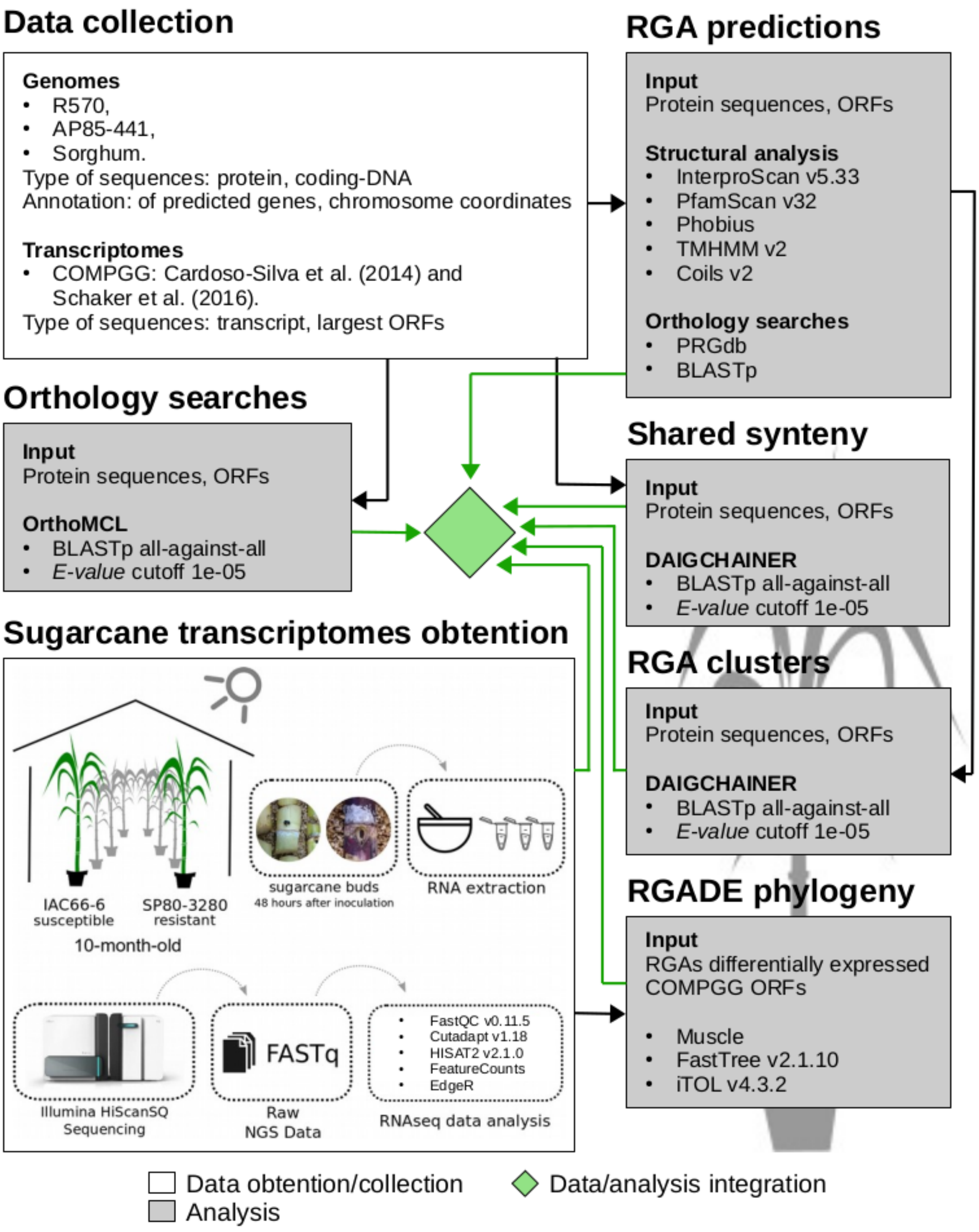

Supplementary Figure S7. Experimental design used in this work. Black arrows indicate the overflow, whereas green arrows indicate how the generated data was integrated for genomic comparisons.

Supplementary Table S1. General RNAseq data statistics used in this study.

Biological replicate	Number of raw paired-end reads	Number of paired-end reads after CutAdapt filtering	Pre-processed reads (%)
IAC_48h_control_R1_C1_S10_L002_R1_001	13,321,602	13,008,288	97.65
IAC_48h_control_R2_C2_S11_L002_R1_001	9,805,246	9,629,952	98.21
IAC_48h_control_R3_C3_S12_L002_R1_001	9,153,047	8,968,846	97.99
IAC_48h_inoc_R1_C4_S13_L002_R1_001	2,724,136	2,594,261	95.23
IAC_48h_inoc_R2_C5_S14_L002_R1_001	7,495,174	7,297,868	97.37
IAC_48h_inoc_R3_C6_S15_L002_R1_001	8,252,851	8,112,783	98.3
SP_48h_control_R1_C7_S16_L002_R1_001	7,857,985	7,631,342	97.12
SP_48h_control_R2_C8_S17_L002_R1_001	6,786,732	6,604,681	97.32
SP_48h_control_R3_C9_S18_L002_R1_001	10,051,357	9,767,445	97.18
SP_48h_inoc_R1_C10_S19_L002_R1_001	9,062,757	8,896,637	98.17
SP_48h_inoc_R2_C11_S20_L002_R1_001	11,529,523	11,262,410	97.68
SP_48h_inoc_R3_C12_S21_L002_R1_001	9,929,794	9,747,096	98.16

Supplementary Table S2. Overview of the proportion of predicted RGAs and RGADEs in the chromosomes in relation to the total of correspondent predictions within each of three sugarcane genome references.

Chr	R570			AP85-441			*S. bicolor*		
	Total RGA	IAC66-6 RGADE	SP80-3280 RGADE	Total RGA	IAC66-6 RGADE	SP80-3280 RGADE	Total RGA	IAC66-6 RGADE	SP80-3280 RGADE
1	0.14	0.07*	0.07**	0.12	0.08*	0.08**	0.13	0.07**	0.08**
2	0.11	0.09	0.11	0.17	0.14	0.17	0.14	0.12	0.13
3	0.11	0.13	0.12	0.11	0.10	0.07**	0.12	0.12	0.09
4	0.11	0.12	0.12	0.09	0.09	0.12	0.08	0.08	0.09
5	0.10	0.19**	0.16*	0.15	0.22**	0.19*	0.12	0.24**	0.19**
6	0.08	0.07	0.06	0.14	0.14	0.15	0.07	0.04*	0.05
7	0.10	0.06	0.08	0.10	0.12	0.11	0.07	0.05	0.07
8	0.06	0.09	0.10	0.07	0.07	0.07	0.07	0.12**	0.11
9	0.07	0.01*	0.06				0.07	0.06	0.06
10	0.08	0.12	0.08				0.08	0.06	0.07

Proportions in relation to the total are different and significant at the level of $P < 0.01$ (**) or $P < 0.05$ (*) by the Fisher's exact test.
